# Supplementary material for: The composition, geography, biology and assembly of the coastal flora of the Cape Floristic Region
Source: PeerJ. 2021 Aug 11;9:e11916. doi: 10.7717/peerj.11916 (PMC8364326; doi:10.7717/peerj.11916)
Supplement: Supplemental Information 2 [file peerj-09-11916-s002.docx]

**Table S1:** Ranking of the 20 most speciose families and genera in the coastal flora (1,365 spp. total) of the Cape Floristic Region.

| **Largest families** | | | | **Largest genera** | |
| --- | --- | --- | --- | --- | --- |
| **Family** | **No. species** | **No. genera** | **Species/genus** | **Genus** | **No. species** |
| Asteraceae | 198 | 53 | 3.7 | *Erica* | 28 |
| Fabaceae | 103 | 22 | 4.7 | *Aspalathus* | 28 |
| Iridaceae | 76 | 16 | 4.8 | *Agathosma* | 26 |
| Rutaceae | 61 | 9 | 6.8 | *Senecio* | 25 |
| Scrophulariaceae | 61 | 13 | 4.7 | *Helichrysum* | 24 |
| Aizoaceae | 59 | 19 | 3.1 | *Indigofera* | 23 |
| Poaceae | 53 | 30 | 1.8 | *Hermannia* | 22 |
| Cyperaceae | 47 | 14 | 3.4 | *Ficinia* | 19 |
| Hyacinthaceae | 30 | 10 | 3.0 | *Moraea* | 19 |
| Apiaceae | 29 | 15 | 1.9 | *Crassula* | 18 |
| Ericaceae | 28 | 1 | 28.0 | *Muraltia* | 18 |
| Polygalaceae | 26 | 2 | 13.0 | *Pelargonium* | 14 |
| Restionaceae | 26 | 6 | 4.3 | *Gladiolus* | 14 |
| Crassulaceae | 25 | 4 | 6.2 | *Phylica* | 14 |
| Malvaceae | 24 | 3 | 8.0 | *Thesium* | 14 |
| Orchidaceae | 24 | 10 | 2.4 | *Asparagus* | 13 |
| Santalaceae | 20 | 4 | 5.0 | *Wahlenbergia* | 12 |
| Amaryllidaceae | 19 | 10 | 1.9 | *Oxalis* | 12 |
| Asphodelaceae | 19 | 8 | 2.4 | *Diosma* | 12 |
| Rubiaceae | 18 | 11 | 1.6 | *Felicia* | 11 |
