# Supplementary material for: The composition, geography, biology and assembly of the coastal flora of the Cape Floristic Region
Source: PeerJ. 2021 Aug 11;9:e11916. doi: 10.7717/peerj.11916 (PMC8364326; doi:10.7717/peerj.11916)
Supplement: Supplemental Information 3 [file peerj-09-11916-s003.docx]

**Table S2:** Ranking of the 20 most speciose families and genera in the calcicole flora (548 spp. total) of the Cape Floristic Region.

| **Family** | **No. spp.** | **Genus** | **No. spp.** |
| --- | --- | --- | --- |
| Asteraceae | 82 | *Erica* | 24 |
| Fabaceae | 46 | *Agathosma* | 19 |
| Aizoaceae | 41 | *Aspalathus* | 16 |
| Rutaceae | 41 | *Senecio* | 12 |
| Iridaceae | 27 | *Indigofera* | 12 |
| Ericaceae | 24 | *Muraltia* | 12 |
| Scrophulariaceae | 24 | *Hermannia* | 10 |
| Apiaceae | 18 | *Helichrysum* | 9 |
| Poaceae | 18 | *Phylica* | 9 |
| Polygalaceae | 16 | *Limonium* | 8 |
| Restionaceae | 16 | *Searsia* | 7 |
| Hyacinthaceae | 12 | *Felicia* | 7 |
| Proteaceae | 11 | *Thamnochortus* | 7 |
| Cyperaceae | 10 | *Wahlenbergia* | 6 |
| Malvaceae | 10 | *Ficinia* | 6 |
| Rhamnaceae | 9 | *Gladiolus* | 6 |
| Plumbaginaceae | 8 | *Pentameris* | 6 |
| Anacardiaceae | 7 | *Diosma* | 6 |
| Campanulaceae | 7 | *Euchaetis* | 6 |
| Caryophyllaceae | 7 | *Delosperma* | 5 |
