# Supplementary material for: The composition, geography, biology and assembly of the coastal flora of the Cape Floristic Region
Source: PeerJ. 2021 Aug 11;9:e11916. doi: 10.7717/peerj.11916 (PMC8364326; doi:10.7717/peerj.11916)
Supplement: Supplemental Information 4 — Tallies are for: the calcicole-ubiquist flora (calcicolous species occurring on both dunes and calcarenites); the calcarenite-endemic flora; and the dune-endemic flora. The total flora comprises 1,365 species. [file peerj-09-11916-s004.docx]

**Table S3:** Number of species in the 20 most speciose families and genera recorded in three subsets of the calcicole flora of the Cape Floristic Region: the calcicole-ubiquist flora (calcicolous species occurring on both dunes and calcarenites); the calcarenite-endemic flora; and the dune-endemic flora. The total flora comprises 1,365 species.

| **Calcicole-ubiquist (104 spp. total)** | | | | **Calcarenite-endemic (218 spp. total)** | | | | **Dune-endemic (226 spp. total)** | | | |
| --- | --- | --- | --- | --- | --- | --- | --- | --- | --- | --- | --- |
| **Family** | **No. spp.** | **Genus** | **No. spp.** | **Family** | **No. spp.** | **Genus** | **No. spp.** | **Family** | **No. spp.** | **Genus** | **No. spp.** |
| Asteraceae | 10 | *Searsia* | 5 | Asteraceae | 28 | *Erica* | 19 | Asteraceae | 44 | *Senecio* | 8 |
| Aizoaceae | 9 | *Agathosma* | 5 | Rutaceae | 27 | *Aspalathus* | 12 | Aizoaceae | 22 | *Helichrysum* | 7 |
| Fabaceae | 9 | *Limonium* | 4 | Fabaceae | 26 | *Agathosma* | 10 | Poaceae | 14 | *Wahlenbergia* | 4 |
| Restionaceae | 7 | *Dasispermum* | 3 | Ericaceae | 19 | *Muraltia* | 7 | Fabaceae | 11 | *Indigofera* | 4 |
| Rutaceae | 6 | *Cynanchum* | 3 | Iridaceae | 13 | *Indigofera* | 6 | Iridaceae | 9 | *Lessertia* | 4 |
| Anacardiaceae | 5 | *Aspalathus* | 3 | Scrophulariaceae | 11 | *Phylica* | 6 | Hyacinthaceae | 8 | *Pelargonium* | 4 |
| Apiaceae | 5 | *Restio* | 3 | Aizoaceae | 10 | *Hermannia* | 5 | Rutaceae | 8 | *Albuca* | 4 |
| Iridaceae | 5 | *Thamnochortus* | 3 | Proteaceae | 10 | *Diosma* | 5 | Scrophulariaceae | 8 | *Hermannia* | 4 |
| Scrophulariaceae | 5 | *Cliffortia* | 3 | Polygalaceae | 9 | *Euchaetis* | 5 | Apiaceae | 6 | *Limonium* | 4 |
| Plumbaginaceae | 4 | *Jamesbrittenia* | 3 | Restionaceae | 8 | *Euryops* | 4 | Cyperaceae | 5 | *Pentameris* | 4 |
| Apocynaceae | 3 | *Lampranthus* | 2 | Apiaceae | 7 | *Felicia* | 4 | Asphodelaceae | 4 | *Agathosma* | 4 |
| Euphorbiaceae | 3 | *Sarcocornia* | 2 | Rhamnaceae | 6 | *Metalasia* | 4 | Campanulaceae | 4 | *Passerina* | 4 |
| Polygalaceae | 3 | *Helichrysum* | 2 | Malvaceae | 5 | *Thamnochortus* | 4 | Caryophyllaceae | 4 | *Delosperma* | 3 |
| Rosaceae | 3 | *Stoebe* | 2 | Lobeliaceae | 4 | *Adenandra* | 4 | Celastraceae | 4 | *Mesembryanthemum* | 3 |
| Amaranthaceae | 2 | *Euclea* | 2 | Poaceae | 4 | *Centella* | 3 | Geraniaceae | 4 | *Capnophyllum* | 3 |
| Cyperaceae | 2 | *Erica* | 2 | Campanulaceae | 3 | *Osteospermum* | 3 | Malvaceae | 4 | *Amellus* | 3 |
| Ebenaceae | 2 | *Adenocline* | 2 | Caryophyllaceae | 3 | *Senecio* | 3 | Plumbaginaceae | 4 | *Felicia* | 3 |
| Ericaceae | 2 | *Indigofera* | 2 | Cyperaceae | 3 | *Ficinia* | 3 | Polygalaceae | 4 | *Gazania* | 3 |
| Myricaceae | 2 | *Otholobium* | 2 | Hyacinthaceae | 3 | *Moraea* | 3 | Thymelaeaceae | 4 | *Silene* | 3 |
| Zygophyllaceae | 2 | *Morella* | 2 | Thymelaeaceae | 3 | *Lobelia* | 3 | Amaranthaceae | 3 | *Erica* | 3 |
