# Supplementary material for: The composition, geography, biology and assembly of the coastal flora of the Cape Floristic Region
Source: PeerJ. 2021 Aug 11;9:e11916. doi: 10.7717/peerj.11916 (PMC8364326; doi:10.7717/peerj.11916)
Supplement: Supplemental Information 5 — Only native flowering plant species were included in the tallies. Data are from: Devil’s Peak, Cowan & Anderson (2014); Kykoedie, Curtis (2013); Agter-Groeneberg Conservancy, Walton (2006). [file peerj-09-11916-s005.docx]

**Table S4:** Number of species in the 10 most speciose families and genera recorded in three site-scale renosterveld floras in the Cape Floristic Region. Only native flowering plant species were included in the tallies. Data are from: Devil’s Peak, Cowan & Anderson (2014); Kykoedie, Curtis (2013); Agter-Groeneberg Conservancy, Walton (2006).

| **Devil’s Peak, Cape Peninsula (103 spp. total)** | | | | **Kykoedie, Overberg (146 spp. total)** | | | | **Agter-Groeneberg Conservancy, Swartland (348 spp. total)** | | | |
| --- | --- | --- | --- | --- | --- | --- | --- | --- | --- | --- | --- |
| **Family** | **No. spp.** | **Genus** | **No. spp.** | **Family** | **No. spp.** | **Genus** | **No. spp.** | **Family** | **No. spp.** | **Genus** | **No. spp.** |
| Asteraceae | 21 | *Oxalis* | 7 | Asteraceae | 16 | *Aspalathus* | 8 | Asteraceae | 46 | *Moraea* | 14 |
| Fabaceae | 12 | *Aspalathus* | 5 | Fabaceae | 14 | *Hermannia* | 7 | Iridaceae | 40 | *Oxalis* | 14 |
| Poaceae | 9 | *Pelargonium* | 4 | Iridaceae | 14 | *Moraea* | 4 | Poaceae | 22 | *Aspalathus* | 12 |
| Oxalidaceae | 7 | *Searsia* | 3 | Aizoaceae | 10 | *Drosanthemum* | 3 | Aizoaceae | 20 | *Lampranthus* | 10 |
| Cyperaceae | 5 | *Senecio* | 3 | Hyacinthaceae | 10 | *Helichrysum* | 3 | Hyacinthaceae | 18 | *Trachyandra* | 7 |
| Geraniaceae | 5 | *Moraea* | 3 | Malvaceae | 9 | *Crassula* | 3 | Fabaceae | 17 | *Helichrysum* | 7 |
| Iridaceae | 5 | *Asparagus* | 2 | Scrophulariaceae | 8 | *Pelargonium* | 3 | Scrophulariaceae | 16 | *Crassula* | 7 |
| Anacardiaceae | 3 | *Helichrysum* | 2 | Poaceae | 6 | *Drimia* | 3 | Oxalidaceae | 14 | *Pelargonium* | 6 |
| Apiaceae | 3 | *Ficinia* | 2 | Amaryllidaceae | 5 | *Ornithogalun* | 3 | Asphodelaceae | 10 | *Lachenalia* | 6 |
| Scrophulariaceae | 3 | *Isolepis* | 2 | Cyperaceae | 5 | *Gethyllis* | 2 | Cyperaceae | 9 | *Drosera* | 2 |
